# Pheno4D: A spatio-temporal dataset of maize and tomato plant point clouds for phenotyping and advanced plant analysis

**DOI:** 10.1371/journal.pone.0256340

**Published:** 2021-08-18

**Authors:** David Schunck, Federico Magistri, Radu Alexandru Rosu, André Cornelißen, Nived Chebrolu, Stefan Paulus, Jens Léon, Sven Behnke, Cyrill Stachniss, Heiner Kuhlmann, Lasse Klingbeil

**Affiliations:** 1 Geodesy Lab, University of Bonn, Bonn, Germany; 2 Photogrammetry and Robotics Lab, University of Bonn, Bonn, Germany; 3 Autonomous Intelligent Systems Lab, University of Bonn, Bonn, Germany; 4 Institute of Sugar Beet Research, University of Göttingen, Göttingen, Germany; 5 INRES Plant Breeding, University of Bonn, Bonn, Germany; Institut de Robotica i Informatica Industrial, SPAIN

## Abstract

Understanding the growth and development of individual plants is of central importance in modern agriculture, crop breeding, and crop science. To this end, using 3D data for plant analysis has gained attention over the last years. High-resolution point clouds offer the potential to derive a variety of plant traits, such as plant height, biomass, as well as the number and size of relevant plant organs. Periodically scanning the plants even allows for performing spatio-temporal growth analysis. However, highly accurate 3D point clouds from plants recorded at different growth stages are rare, and acquiring this kind of data is costly. Besides, advanced plant analysis methods from machine learning require annotated training data and thus generate intense manual labor before being able to perform an analysis. To address these issues, we present with this dataset paper a multi-temporal dataset featuring high-resolution registered point clouds of maize and tomato plants, which we manually labeled for computer vision tasks, such as for instance segmentation and 3D reconstruction, providing approximately 260 million labeled 3D points. To highlight the usability of the data and to provide baselines for other researchers, we show a variety of applications ranging from point cloud segmentation to non-rigid registration and surface reconstruction. We believe that our dataset will help to develop new algorithms to advance the research for plant phenotyping, 3D reconstruction, non-rigid registration, and deep learning on raw point clouds. The dataset is freely accessible at https://www.ipb.uni-bonn.de/data/pheno4d/.

## 1 Introduction

Studying growth processes of plants plays an essential role in modern agriculture and has a long history in research. Mostly, these studies rely on manual measurements and human assessment of growth stages in the field. This approach is time-consuming and prone to human bias. Another popular method for quantitatively and qualitatively studying plants is based on RGB or spectral imaging. This approach has the advantage that it can measure a high number of plants in a short time while also having a non-destructive nature and being less susceptible to human bias. Automated analysis of phenotyping traits of plants using image techniques has become a standard tool. However, these systems suffer from the limitations of the two-dimensional image plane.

Over the last decades, measuring three-dimensional surface information from plants got increasingly popular in phenotyping and agricultural applications. Concerning measuring plants in 3D, laser scanning has the advantages of a high resolution, a high accuracy, and direct access to the scanning object. The 3D point clouds obtained from a laser scanning system describe the plant geometry accurately and can be used to detect subtle changes of plant organs when performing subsequent measurements of the same plant. Recent studies show that measuring phenotypic traits from 3D data yield accurate results [1]. Combining accurate traits estimation with non-destructive methods is attractive in plant phenotyping, thus this field is getting more and more popular with research trying to measure different traits, ranging from internode length [2] and stem diameter to leaf area [3–5].

One can generate even more useful information by monitoring the plants and the above-mentioned traits over time to analyze plant growth and health [4, 6, 7]. Such applications mostly rely on high-resolution point clouds of a certain number of plants measured at different times. Nonetheless, acquiring this data can be tedious due to several reasons. First, a high-resolution 3D laser scanning system is costly and the measuring process is time-consuming. Additionally, some advanced plant analysis methods require annotated training data which is also time-consuming and requires skilled annotators.

**Fig 1:**
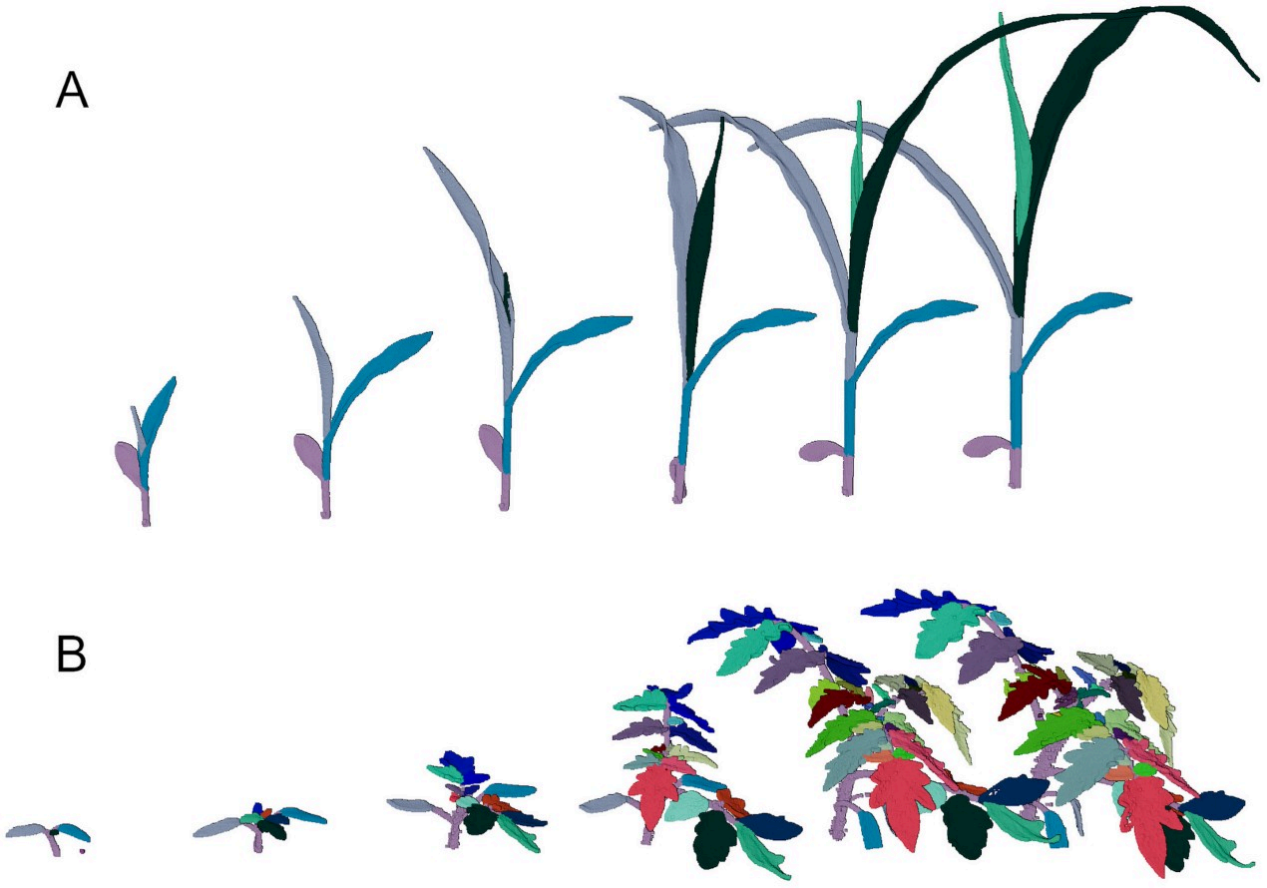
Sample data of a maize (A) and a tomato plant (B) scanned periodically. Temporally consistent labels are assigned to each individual leaf, as indicated by color.

To address these issues, we provide a multi-temporal dataset of 3D point clouds of maize (7 plants, 12 days) and tomato plants (7 plants, 20 days). We raised them in pots in a greenhouse growing station. Shortly after developing the first sprout, we measured them daily with a highly accurate 3D laser scanning system with a spatial precision of less than a tenth of a millimeter. The measurement period lasted for about two weeks for the maize and three weeks for the tomato plants. We manually labeled the point clouds into ‘soil’, ‘stem’, and ‘leaf’ points, where each leaf is assigned a unique label for the whole measuring period. In Fig 1 we show examples of maize and tomato plants captured over two weeks, together with temporally consistent labels.

The contribution of this paper is a large and freely available dataset featuring highly accurate and registered point clouds of 7 maize and 7 tomato plants collected on different days containing approximately 260 million 3D points, scanned at high frequency and precision. In total, these are 49 labeled point clouds of maize plants and 77 labeled point clouds of tomato plants. Together with the point cloud data, we provide temporally consistent manually created labels for each point in the clouds. We also show baseline results, using this dataset for semantic segmentation, instance segmentation, and surface reconstruction as well as some derived time series of leaf area, leaf length, and stem length.

This article is organized as follows. We substantiate the importance of large-scale datasets in computer vision in Sec. 2. We also point out how a sub-millimeter accuracy 3D dataset of plants will be beneficial for the community in the agricultural field. We then briefly review the potential of our dataset for specific open problems in phynotyping based on computer vision techniques. In Sec. 3, we detail the data acquisition process. This covers the laser scanning system, the measurement procedure, the data pre-processing, and the point cloud labeling. We also quantify the amount of provided data. In Sec. 4, we then highlight the usability of the data by showing four exemplary use cases with applications from which we believe the data will find use in. Finally, we calculate phenotypic traits to underline the capability of the dataset to track plant organs over time based on its traits such as leaf length.

## 2 Related work

Datasets and benchmarks have always driven the progress of computer vision research [8]. The availability of large-scale datasets, such as ImageNet [9], was a fundamental prerequisite for the emergence of deep learning as we know it today. In the following, computer vision researchers established a diverse number of challenges [10, 11] that drove the progress of the entire community. However, those challenges are designed to develop algorithms on 2D images while datasets and benchmarks for 3D data are comparably fewer.

3D datasets gained interest in recent years especially in tasks related to robot navigation and autonomous driving. On one hand, such datasets contain images from RGB-D sensors [12–15] but also synthetically generated images [16]. On the other hand, the intensive research on autonomous driving lead to the publication of several datasets [17–22] among which the KITTI Vision Benchmark [23], and its annotated version [24], are the biggest ones. In the context of human activity recognition, several datasets have been published that are specially designed for mesh registration, both rigid and non-rigid [25, 26]. Additionally, ShapeNet [27] is especially noteworthy for point clouds showing a single object. However, such data is not directly transferable to other domains.

Agricultural datasets for computer vision tasks follow a similar evolution. In the beginning, researchers provided datasets of 2D images with labels for 2D tasks such as semantic segmentation. The images came from robotics platforms, both ground [28] and aerial [29], or were synthetically generated through computer graphics engines [30, 31]. Chebrolu et al. [32] presented a larger robotic dataset with measurements from a diverse number of sensors, including odometry for robot navigation and RGB images. Recently, two datasets of plant point clouds were released. Khanna et al. [33] presented a dataset containing biweekly color images, infrared stereo image pairs, and multispectral camera images along with applied treatments and weather conditions of the surroundings. The resulting point clouds are only top-down views of the plants, providing 2.5D information rather than 3D models. Point clouds taken with the utilized sensor have a millimeter-level accuracy [34]. Dutagaci et al. [35] presented a dataset of 11 annotated 3D models of real rosebush plants acquired through X-ray tomography both in volumetric form and as point clouds. The individual X-ray images have a slice spacing of 0.5 mm and a pixel spacing of about 1 mm.

Comparisons [5] between state of the art systems for 3D measuring of plant traits—as used in plant phenotyping—show that laser triangulation scanners not only provide the highest accuracy on sub-millimeter level but their point clouds are also found to be a well-suited input for machine learning methods. Other 3D measuring methods such as structured light approaches, structure from motion, time of flight, and light field measuring only offer an accuracy and resolution in the order of millimeters. Dupuis and Kuhlmann [36] performed an uncertainty evaluation of the same triangulation-based scanner we used for our plant dataset. The results show a dependence of measurement accuracy on the adaption of exposure time and surface structure. However, the measurement accuracy always was well below 1 mm.

To the best of our knowledge, our dataset is the first one featuring highly accurate point clouds of plants, with sub-millimeter accuracy (∼ 0.1 mm), acquired daily over an extended period of time. In Table 1 we show a comparison between our dataset and the datasets featuring 3D point clouds of plants [33, 35].

**Table 1:** Our dataset contains a considerably higher number of point clouds with respect to other public datasets. Note that the ETH dataset is meant to develop algorithms to evaluate plant stress.

Dataset	Point Clouds	Extension Period	Measure Frequency	Sensor	Labels
Rose-X [35]	11	single day	-	x-ray tomography	semantic
ETH [33]	16	2 months	bi-weekly	RGB-D + HSI	applied treatments
Pheno4D (ours)	126	2–3 weeks	each 2 days	laser	plant organ instances

The availability of datasets and benchmarks is crucial for empirical evaluation of research for at least three reasons: (i) providing a basis to measure progress, by reproducing and comparing results, (ii) uncovering shortcomings of the current state of the art and therefore paving the way for novel approaches and research directions, and (iii) enabling to develop approaches without the need to first collect and label data. In the following, we briefly review open problems in phenotyping based on computer vision techniques where we believe that this dataset will be beneficial.

**Instance segmentation** is the task of densely labeling the input into disjoint regions corresponding to distinct objects of interest. Recent advances in deep learning lead to different methods to process raw unorganized point clouds, either by processing each point individually [37, 37] or by defining convolutions in the 3D space [38, 39]. In the context of plant phenotyping, it is interesting to segment distinct instances of plant organs for the purposes of computing phenotypic attributes for each plant. Gaillard et al. [40] operates on voxel data and performs thinning to extract a skeleton which is then used for segmentation of leaves and stem. It requires learning from a dataset of manually labeled plant skeletons. Le Louedec et al. [41] detects broccoli heads in point cloud data. However, they require the cloud data to be organized in an image-like grid which limits their use cases to only RGB-D sensors that capture the scene from a single point of view. Kusuman et al. [42] uses 3D point clouds recorded from a KinectV2 to detect and track broccoli heads in the field. They use a series of handcrafted features and classify the points using a Support Vector Machine (SVM). Shi et al. [43] performs semantic and instance segmentation by processing multiple views of the same plant using a CNN. The 2D predictions are fused in 3D using a voting scheme.

**Surface reconstruction** involves capturing the shape of the object as a continuous representation from the point cloud scans which are discrete, noisy, and often exhibit missing regions. These surfaces can be represented as triangular meshes, set of planes, or other primitive shapes or via certain implicit fields [44]. Surface reconstruction is a popular topic in computer graphics. Several algorithms such as Poisson [45], TSDF [46, 47] etc. have been developed over the last two decades and are widely used. However, applying such techniques to plant data is not trivial because of the structural complexity in the shape of the plants [48]. In [49] the challenges of the reconstruction of leaf surfaces and the influence of the meshing procedure on the leaf area estimation have been demonstrated. Some recent works have looked at these challenges and show promising results such as the work by Yin et al. [50] who have generated high-quality plant reconstructions by cutting out different parts, performing reconstruction separately for each part, and finally assembling them together. Furthermore, Zheng et al. [51] can capture the shape of blooming flowers and track them over time. Despite these advances, surface reconstruction for plants involves designing techniques for specific plant types and often includes a substantial amount of manual interventions. Giving these challenges, surface reconstruction for plants is still an open problem.

**Point cloud registration** techniques such the Iterative Closest Point (ICP) [52, 53] only consider rigid motions, whereas other non-rigid registration techniques are often restricted to articulated motions such as that of human skeletons [54–56] or synthetic models used in animation or computer graphics applications [57–59]. Such techniques often don’t capture the complexities specific to plant growth and are unable to register plant point clouds reliably.

Our dataset consists of point clouds from the surface of maize and tomato plants measured on several days over their growth period. Each point has a label information. These features meet the requirements of computer vision tasks such as instance segmentation, surface reconstruction, as well as point cloud registration. We are convinced that our dataset will contribute to drive the research in the mentioned as well as further areas related to plant phenotyping forward.

## 3 Data acquisition

**Fig 2:**
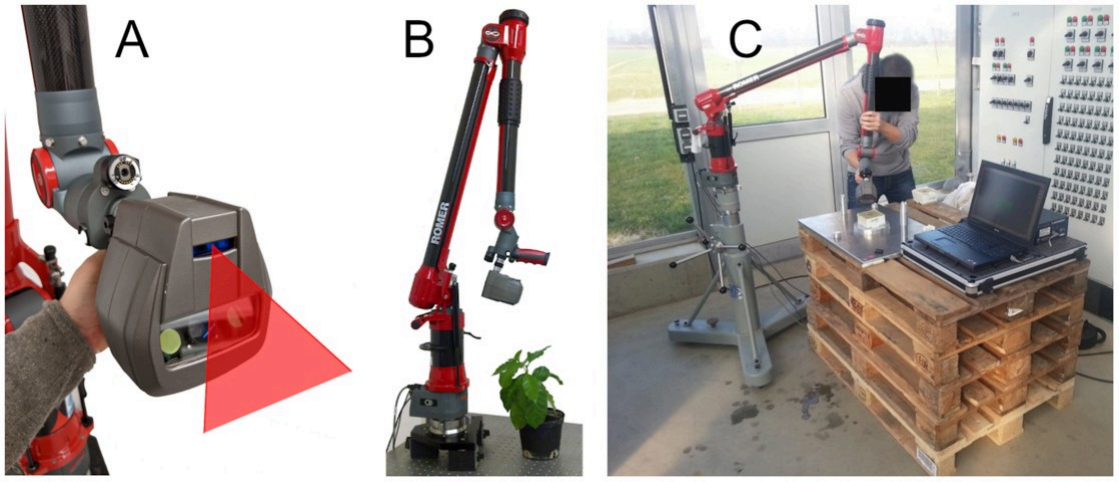
Tomato plants (A) and maize plants (B) in pots in the growing station and the process of measuring with the scanner of a tomato plant (C).

In our measurement campaign, we captured point clouds from 7 maize and 7 tomato plants daily. We started the measurement period shortly after seeing the first sprouts and we captured data for about two weeks for the maize and for about three weeks for the tomato plants. This means that we observed the plants in an early growth stage. Fig 2 shows the plants in their growing environment and gives an impression of the plant stages.

### 3.1 Laser scanning system

**Fig 3:**
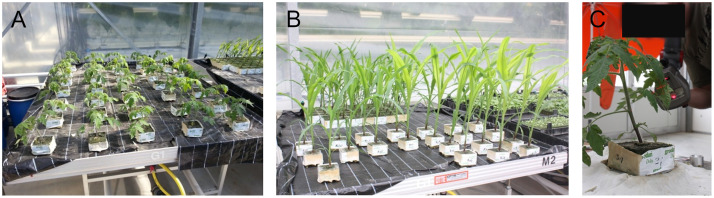
Laser scanning system. Scanning device (A), complete scanning system (B) consisting of the scanning device attached to the measuring arm, and the scanning system in the measurement laboratory environment (C).

The sensor we used for the data acquisition consists of a light section scanner coupled to an articulated measuring arm. The scanning device was a Perceptron Scan Works V5 laser triangulation scanner (Perceptron Inc., Plymouth, MI, USA) as shown in Fig 3. The system was originally developed for the field of industrial quality management and works with a wavelength of 660 nm. The output of the scanner is a 2D scan profile, that has a width of 93-140 mm and the mean measuring distance is 100 mm. The resolution reaches up to 7640 points per scan line at a measuring accuracy of *σ* = 0.012 mm at a measurement frequency of up to 60 profiles per second.

To obtain the position and orientation of the scanner with respect to the plant, it was mounted on a measuring arm, a ROMER Infinite 2.0 by Hexagon Metrology Services Ltd., London, UK. It has a spherical measurement volume with a radius of 1.4 m and consists of seven joints and three links. The arm elements are made out of carbon fiber to ensure geometrical as well as thermal stability. The accuracy of measuring the tip of the arm is 45 *μ*m. Fig 3 shows the measuring arm together with the attached scanning device. Using the very accurate position and orientation information from the arm, the 2D scan profiles from the scanner can be registered to a consistent 3D point cloud in the local coordinate system of the arm.

### 3.2 Measurement procedure

We performed the measurements in the greenhouse, where the plants were grown. We scanned each plant separately, using a mounting system, which ensured, that the pot had the same position and orientation relative to the measuring arm every day. In this way, the plant’s position and orientation were consistent during the whole series of scans. The measuring volume was sufficient to scan the plant from different positions and angles. Due to the non-invasive nature of the scanning system, we were able to carry out the measurements with the motion of the plant. The data acquisition time for each plant was several minutes, depending on the size and complexity of the plant structure. The resulting point cloud of the plant was then given in the local reference frame of the measuring arm.

**Fig 4:**
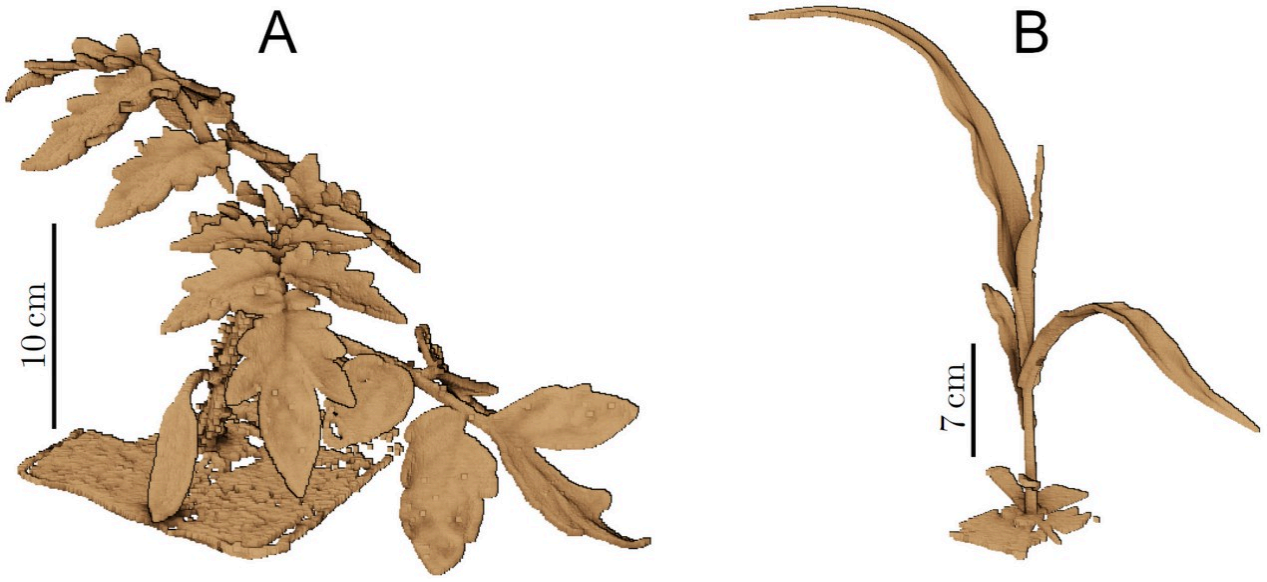
Raw point clouds of a tomato plant (A) and a maize plant (B). For illustration purpose the point size has been increased in the visualization.

Fig 4 shows the resulting point clouds of a tomato plant and a maize plant. One can observe that the structure of the tomato plant is significantly more complex. Consequently, the scanning process for a tomato plant was longer than for a maize plant, regardless of the growing stage of the plants in this measurement campaign. For example, a tomato plant measured at the more advanced stage of the measuring period takes about 15 minutes while a maize plant at a later stage of the experiment can be measured in less than 10 minutes. Two key properties that we ensured, are the complete coverage of the plant surface and the stillness of the plants during the measurements. Despite the efforts, some point clouds still exhibit small areas with missing measurements, for example, due to occlusion.

### 3.3 Data pre-processing

Point cloud pre-processing only consisted of a manual outlier removal step. Points that could not be considered as part of the plant or soil were considered as outliers. Besides that, the point clouds were not preprocessed in another way, i.e. the point clouds still include parts of the pot and the soil. In case the inhomogeneous point distribution of the provided point clouds leads to problems in further data processing such as meshing, a homogenization of the data still can easily be done with any point cloud processing software.

### 3.4 Point cloud labeling

To use this dataset for plant analysis tasks, we provide labels for each 3D point in the dataset. We labeled each point as ‘soil’, ‘stem’, or ‘leaf’ point. Furthermore, each leaf receives its unique label, making it distinctive from the other leaves on the same plant. The label of a particular leaf is the same for consecutive point clouds of this plant and is consequently consistent for the whole series of scans. In the following, we explain the procedures we used for labeling, and some issues, which emerged in the process.

#### 3.4.1 Labeling tomato plants

**Fig 5:**
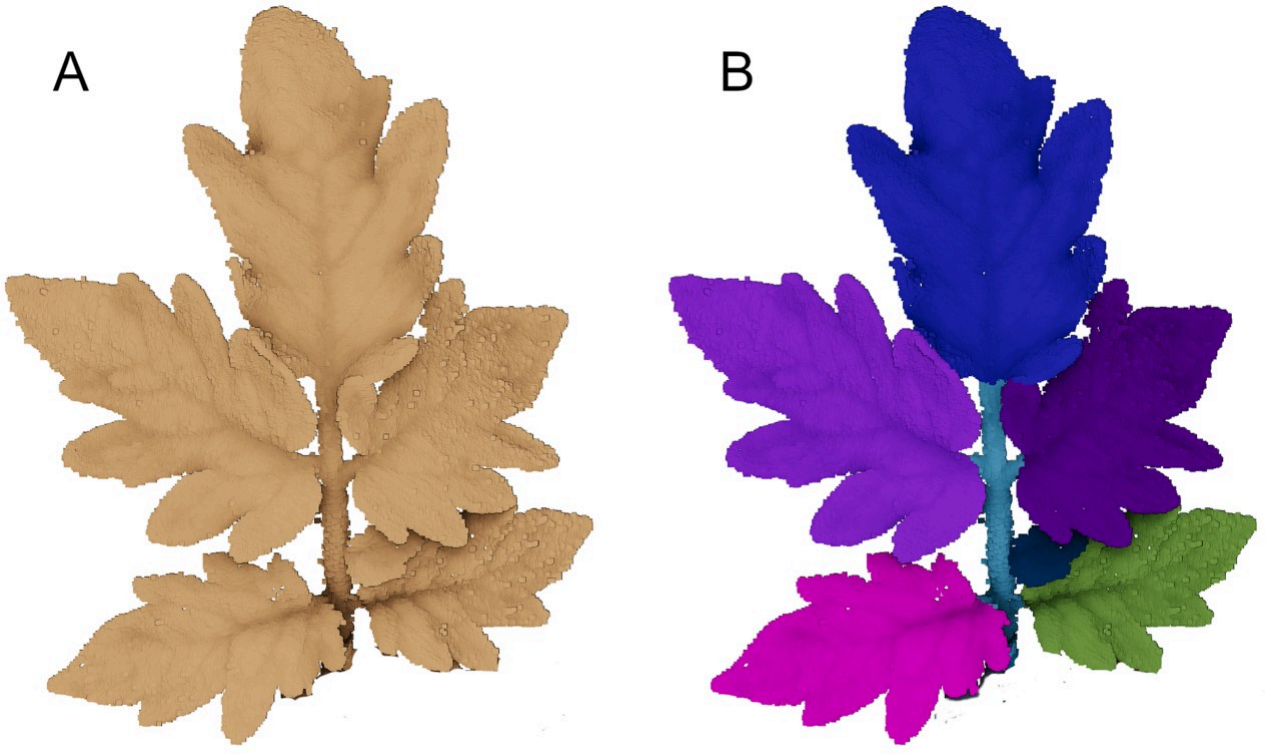
Detailed view of a tomato plant point cloud (A) and its segmentation into stem and individual leaves, illustrated in different colors (B).

Fig 5 shows a detailed view of a raw tomato plant point cloud as well as its segmentation into individual leaves and the stem. It can be seen, that the transition from stem to leaf can be detected quite easily at the spot where the leaf starts to spread out.

#### 3.4.2 Labeling maize plants

**Fig 6:**
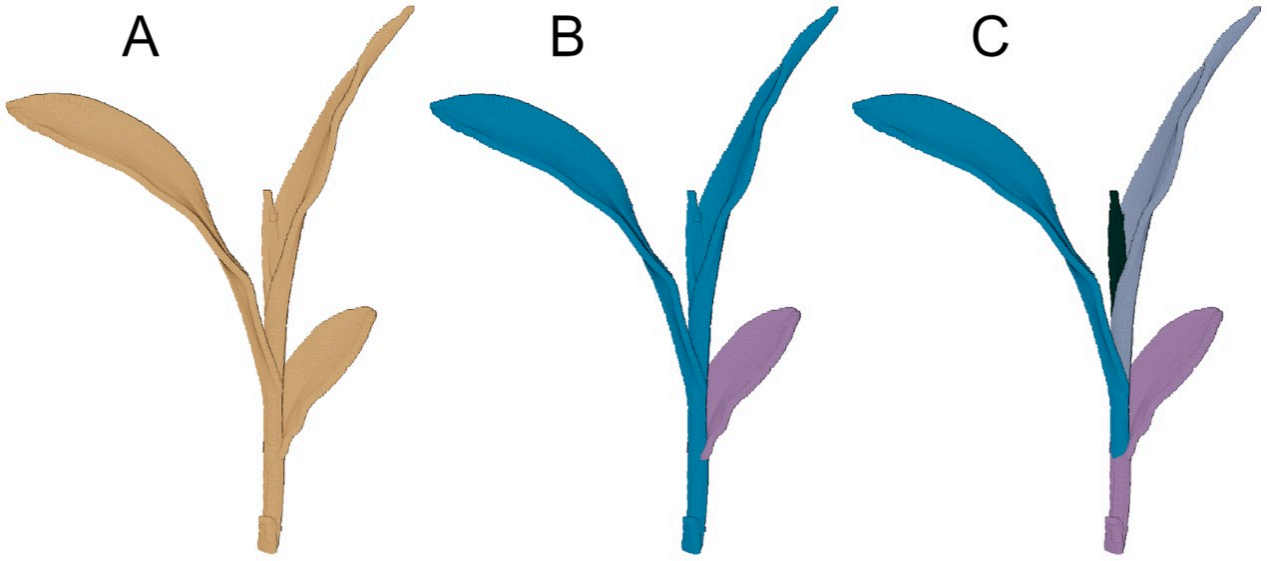
Point cloud of a maize plant (A), segmentations derived according to the Leaf Collar Method (B) and according to the Leaf Tip Method (C).

In contrast to the tomato plants, the separation of the maize plants into stem and leaves is not as obvious because maize plants do not show a clearly identifiable stem. Leaves are emerging from the whorl of the plant without showing a distinctive region that separates the respective leaf from the rest of the plant. Therefore, we labeled the maize point clouds using the two following approaches, which we derived from two commonly used methods for staging maize plants. The approaches are shown in Fig 6. Subfigure Fig 6A shows a point cloud of a maize plant as it has been captured in the measurement series. The first way of labeling is derived from the ‘Leaf Collar Method’ [60]. The resulting segmentation is shown in Fig 6B. The second way of labeling is derived from the ‘Leaf Tip Method’, see Fig 6C. We explain both methods below.

#### 3.4.3 Leaf collar method

**Fig 7:**
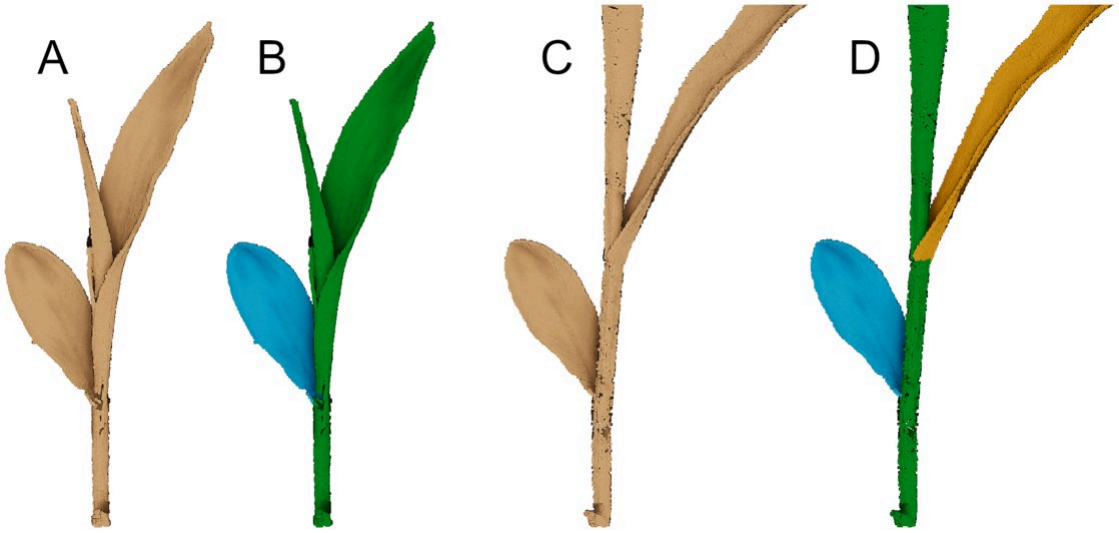
Labeling a maize plant into stem and leaves. Detailed view of a maize point cloud (A) and its segmentation derived according to the Leaf Collar Method (B). The same maize plant two days later (C) and its segmentation derived according to the Leaf Collar Method (D).

Fig 7A shows a detailed view of a point cloud of a maize plant. As it can be seen, there is no distinctive area, which separates the leaf blade on the right-hand side from the stem or rather the rest of the plant. The leaf on the right emerges from the whorl of the plant. Hence, a clear definition is needed of what parts of the point cloud are considered as individual leaf. The Leaf Collar Method is a method for staging corn plants. Crop staging is used to assess crop development and to make recommendations about plant-related applications. This method determines the leaf stage in maize by counting the number of leaves on a plant with visible leaf collars [60]. The leaf collar is the light-colored collar-like “band” located near the spot where the leaf blade comes in contact with the stem of the plant. Leaves, which are not fully expanded and show no visible leaf collar yet are not considered as a leaf in this leaf stage method. Following that, the point cloud from Fig 7A is separated into a leaf on the left (blue) and the stem (green) as shown in Fig 7B. The leaf blades on the top right and top left are not considered as individual leaves yet and therefore still belong to the stem. Fig 7C shows the same plant two days later and Fig 7D shows the labeled point cloud. It can be seen that the leaf on the top right now has its own label. Taking a closer look at the spot where the leaf comes in contact with the stem reveals a clear fold. The leaf on the top left is still considered as part of the stem and gets its own label as soon as a leaf collar is visible. This method leads to a labeling as shown in Fig 6B.

An advantage of this labeling approach is that the labels are compliant with one of the most common maize staging methods [61]. At the same time, this approach can have disadvantages depending on the use of this data. In Fig 7B the two emerging leaf blades on the top are assigned to the stem of the leaf. This way of labeling could lead to problems in regard to later processing steps, for example in automatic point cloud segmentation as diverging parts of the plants have the same label and share similar geometric features to 3D points classified as leaf. Furthermore, leaf blades are affected by label changes. Before a leaf develops a leaf collar and receives its own label, it has the same label as the stem. This happens from one day to another as exemplarily shown in Fig 7.

#### 3.4.4 Labeling derived from the Leaf Tip Method

The second way to label maize point clouds is derived from the Leaf Tip Method. In this method for staging corn plants, simply the leaf tips are counted from the bottom to the top of the plant. Young leaves that are emerging from the whorl are included in the counting. There is no particular label for the stem and except for points labeled as soil, each point is assigned to a leaf. The region for a leaf consists of the leaf blade and the stem part of the plant downwards to the spot where the next leaf blade emerges from the plant. These considerations are regardless of whether a leaf has a visible leaf collar or not. This method leads to a labeling as shown in Fig 6C. This way of labeling prevents the labeled point cloud from having diverging parts that have the same label. Furthermore, label changes for parts of the plant from one day to another do not appear. These conditions could make the labeled point clouds more suitable for subsequent processing steps of the data.

### 3.5 Provided data

**Fig 8:**
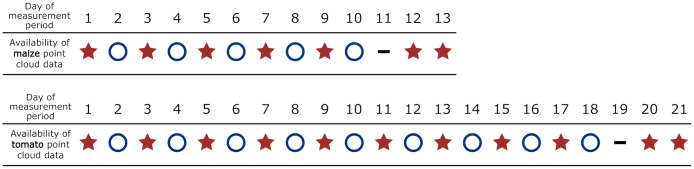
Dataset coverage of the 7 maize plants (top) and the 7 tomato plants (bottom) with respect to the day within the measurement period (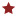 = labeled point clouds available, 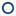 = only point clouds available, 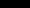 = no data available).

The dataset consists of labeled as well as unlabeled point clouds of 7 maize and 7 tomato plants. The extent of the dataset can be explained on the basis of Fig 8. It shows for which day the point clouds of the 7 maize and 7 tomato plants are available and whether the point clouds are annotated or not. As can be seen, each plant was measured every day except for the eleventh day of the maize measurement period and the nineteenth day of the tomato measurement period. No measurements were conducted on these respective days. The labeling was performed for every second day and even for consecutive days at the end of both measurement periods (days 12 and 13 for the maize period and days 20 and 21 for the tomato period).

The dataset contains a total of 84 maize point clouds (about 90 Mio. points). From these, 49 point clouds (about 60 Mio. points) additionally are labeled with both labeling approaches. Furthermore, the dataset contains a total of 140 tomato plants (about 350 Mio. points) from which 77 point clouds (200 Mio. points) contain labels. The point clouds are provided as human-readable ASCII tables, in which the first three columns represent the coordinates. The fourth column of tomato point cloud files represents the labels. For maize point cloud files, the fourth column represents the labels derived from the Leaf Collar Method and the fifth column the labels derived from the Leaf Tip Method. We provide the data at https://www.ipb.uni-bonn.de/data/pheno4d/.

### 3.6 Software API

We provide an API for accessing the data and loading it in both Python and C++. The data loader can be used to subsample the point clouds, perform data augmentation by transforming it, select specific days and plants to load, and various other functionalities. We provide the code and the examples for loading the data at https://github.com/AIS-Bonn/data_loaders.

## 4 Use cases

In this section, we want to highlight applications in which our dataset can be used as well as providing baselines to speed up future comparisons.

### 4.1 Semantic segmentation

Deriving phenotypic traits like leaf area or stem length from point cloud data requires distinguishing between the leaves, stem, and ground. Semantic segmentation approaches assign a label to each data point and often are machine learning methods that can be trained to segment the data into such classes. Today, most classification and semantic segmentation systems rely on deep learning. See LeCun et al. [62] for an introduction. However, most deep learning for semantic segmentation is applied to images that exhibit a regular grid-like structure, allowing the usage of convolutions to perform feature extraction. Point clouds are unstructured and therefore require specialized methods to be semantically segmented. Here we evaluate several approaches that are capable of segmenting raw point clouds.

**Fig 9:**
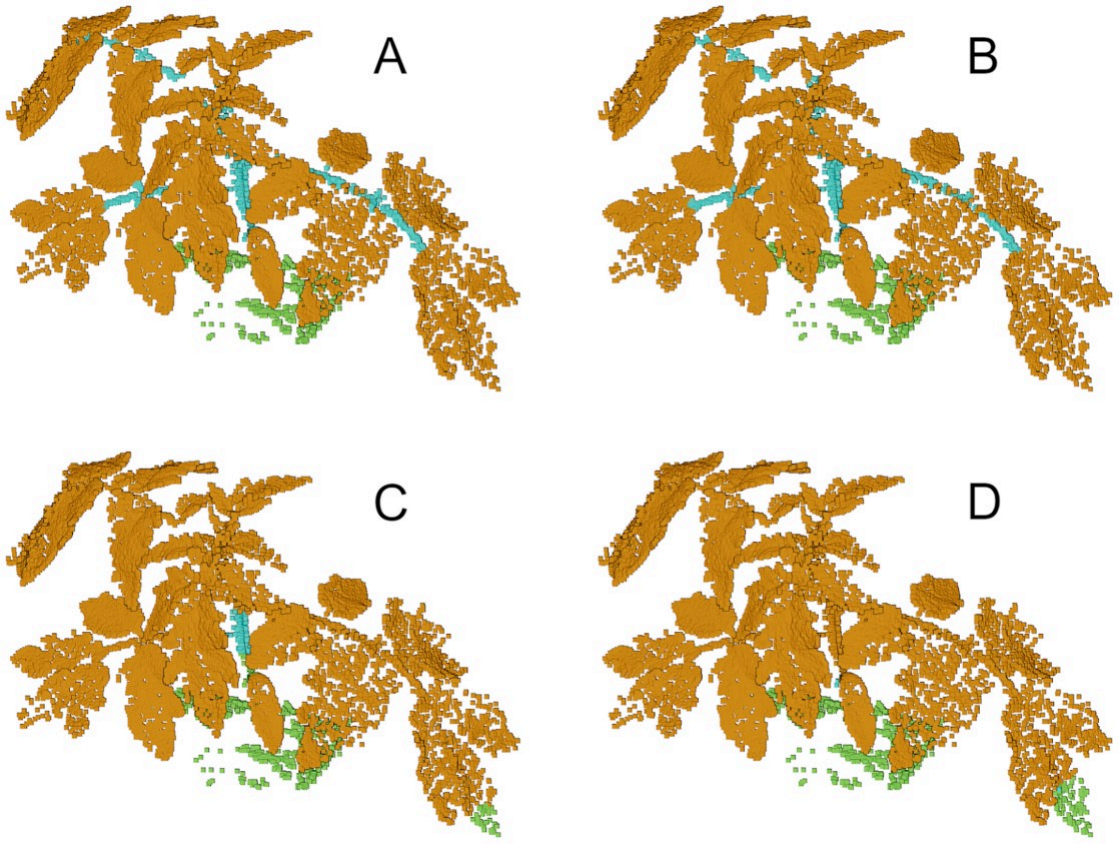
Semantic segmentation comparison between different deep learning approaches (Ground-truth (A), LatticeNet (B), PointNet++ (C) and PointNet (D)). PointNet++ misses parts of the stem and PointNet misses it completely. In contrast, LatticeNet segments the whole stem accurately.

We trained three different neural network architectures (PointNet [37], PointNet++ [37], LatticeNet [38]) for the task of semantically segmenting the raw cloud into leaf, stem and ground. We trained for maize and tomato separately and used 5 plants for training and 2 for testing. During training, we performed data augmentation by randomly translating, rotating, and stretching the clouds. Additionally, the clouds were sub-sampled to approximately 50K points to cope with memory restrictions. For each architecture, we report the mean Intersection-over-Union and the IoU per class in Table 2. The segmentation results are exemplary shown in Fig 9.

**Table 2:** Mean Intersection-over-Union and IoU per class results for three different neural network architectures tested on our maize and tomato point clouds.

	Maize				Tomato			
	mIoU	leaf	stem	ground	mIoU	leaf	stem	ground
PointNet [37]	64.0	92.2	5.9	93.9	64.7	83.9	11.9	98.4
PointNet++ [63]	83.9	95.2	58.3	97.1	68.4	88.8	19.6	98.6
LatticeNet [38]	**98.8**	**99.8**	**97.0**	**99.7**	**95.1**	**98.7**	**86.9**	**99.8**

We observe that all three methods achieve high IoU (>80% IoU) in the leaf and ground class. However, the PointNet-based methods struggle with the stem class which is a relatively small class with few points. LatticeNet achieves good results in all classes. This may due to the fact that LatticeNet applies convolutions on a permutohedral lattice while the PointNet-based methods rely on pooling point features to obtain their internal representation. Convolutions in the lattice space allow the usage of larger spatial context as the lattice size is independent of the number of points in the clouds. In contrast, PointNet-based methods struggle with high-density point clouds as they operate only on the points.

### 4.2 Instance segmentation

Instance segmentation refers to the task of not only assign to every data point a semantic label but also an instance ID. In this way, it becomes possible to distinguish different objects of the same class such as two leaves. Computing more advanced phenotypic traits like leaf angle or leaf length require to explicitly distinguish between each individual leaf in the plant. For this, instance segmentation has to be performed to extract each instance of the leaves.

Using our dataset, we train three different neural network architectures (PointNet [37], PointNet++ [37], LatticeNet [38]) as baselines for the task of instance segmentation, namely classifying each point as stem, ground, or one leaf instance using the same clustering loss in the embedding space as defined by De Brabandere et al. [64]. To facilitate other researchers comparing their approaches to these baselines, we report the Symmetric Best Dice (SBD) [64] score in Table 3.

**Table 3:** Segmentation performance of different deep learning on Pheno4D dataset.

	SBD	
	Maize	Tomato
PointNet [37]	69.7	47.3
PointNet++ [63]	74.8	56.1
LatticeNet [38]	**80.6**	**74.2**

### 4.3 Spatio-temporal point cloud registration

**Fig 10:**
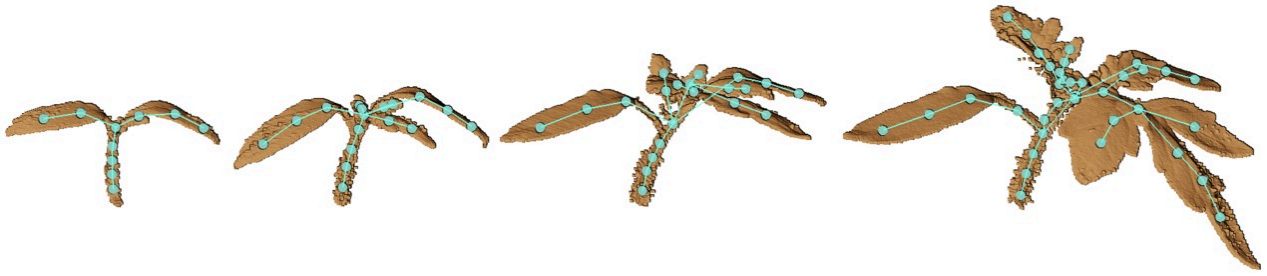
Time series of a tomato plant scanned in various days together with the extracted skeleton.

**Fig 11:**
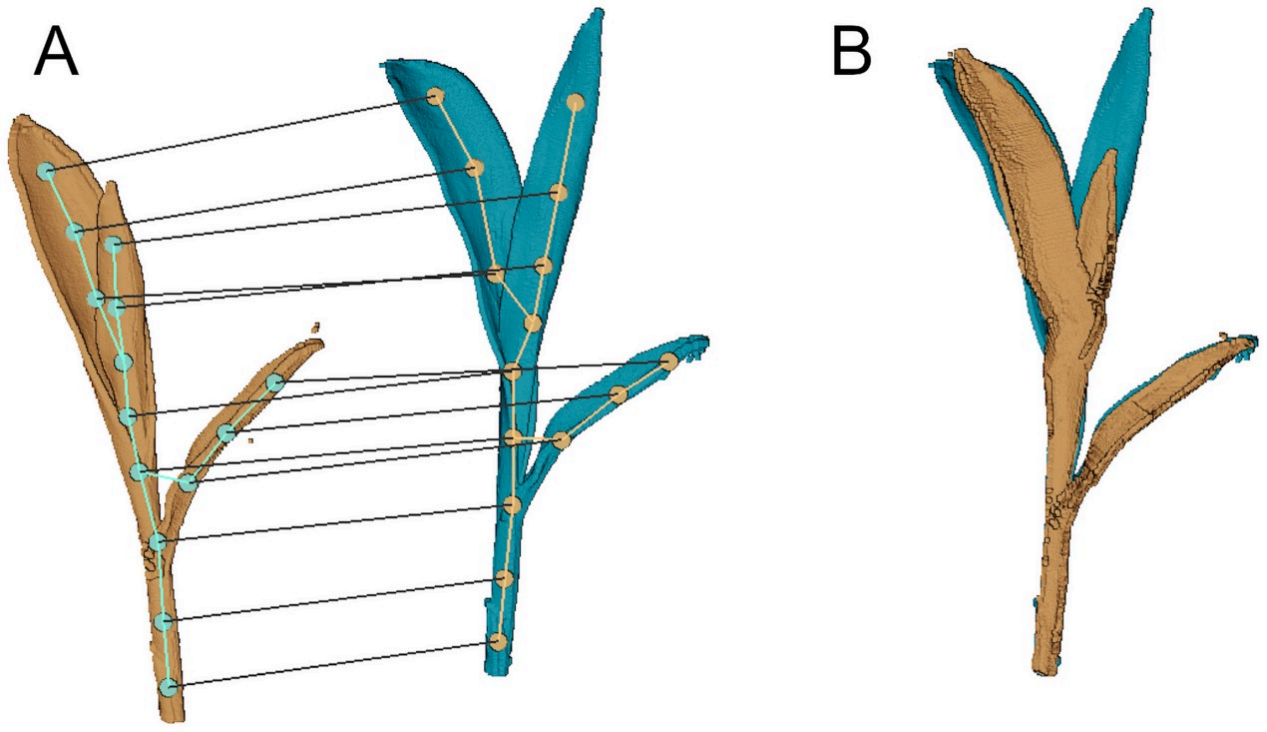
Spatio-temporal registration. Individual organs are segmented and skeletons are fitted (A). The skeletons corresponding to different scanning days are associated temporally. The correspondences allows to non-rigidly deform one cloud in order to match the other (B).

The time-series plant point cloud data we present in this paper will be useful in developing techniques that analyze the plant growth over time. One such application is to track different phenotypic traits for the plant and quantify its performance. In order to perform such temporal analysis, there is a need to come up with techniques that associate the point cloud data over time and register them against each other. However, registration of plants over an extended period of time is challenging due to their changing topology, anisotropic growth, as well as the non-rigid motion in between plant scans. Some progress in this direction has been made in recent works based on a subset of the dataset presented in this paper. To deal with the complexities of registering plant data over time, Chebrolu et al. [7] and Magistri et al. [4] exploit the skeleton structure of the plant to obtain correspondences between the same plant parts for the scans taken on different days (see Fig 10). Using these correspondences, they then estimate a non-rigid registration composed of a chain of local affine transformations that captures the growth as well as bending in the plants effectively. Based on this procedure they obtain encouraging results in terms of registration accuracy as well as demonstrating an application for tracking phenotyping traits. Some example results from the registration process are shown in Fig 11. We believe by making our dataset available to the larger community, we will provide a further push towards developing more robust and efficient techniques for registering point cloud data of plants.

### 4.4 Surface reconstruction

**Fig 12:**
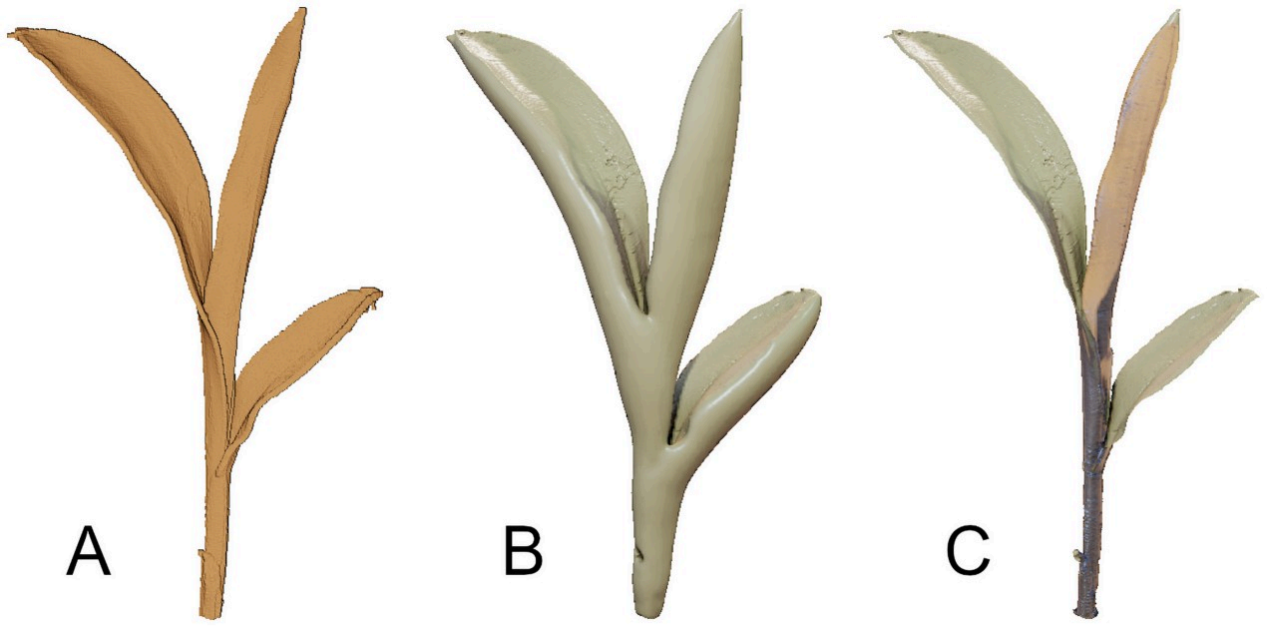
Surface reconstruction. Raw point cloud (A), initial mesh extracted using Poisson reconstruction (B) and mesh after trimming of triangles with low density (C).

Generating smooth surface reconstruction of plant point clouds would play a key role in several phenotyping tasks such as obtaining key traits such as surface area and track them over time. However, off-the-shelf techniques typically fail to produce good reconstructions for plant data, because of the complex shape and topology typical of plants. For example, plants usually contain thin surfaces such as leaves, as well as other thicker regions such as the stem or the branches. Most techniques are unable to handle these different scales, as well as other issues related to estimating reliable normal information for thin structures. A particularly challenging example would be reconstructing extremely fine hair-like structures on the stems of tomato plants. Other complications arise from the self-occlusions that are present in the plant data where the outer leaves occlude the inner structures making them unobservable. All these challenges make the surface reconstruction for plants a challenging task with several open questions and opportunities for developing new techniques to address them. In Fig 12 we show an example of Poisson reconstruction on our dataset. Note that we need to manually clean the resulting mesh to obtain a meaningful representation of the considered plant. We believe that the high-fidelity point cloud dataset we present in this paper would help in the developing and testing of different reconstruction techniques that aim to address the challenges we discussed earlier.

### 4.5 Phenotyping

**Fig 13:**
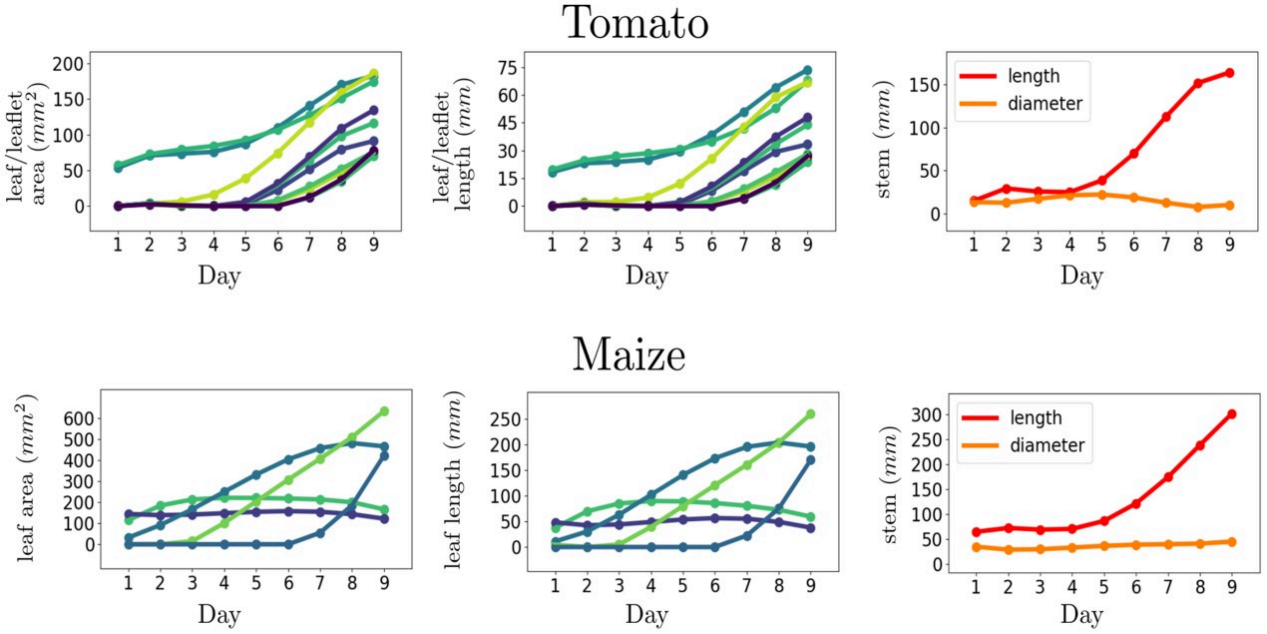
Tracking of phenotyping traits starting from raw point clouds.

Combining the techniques described before, it is possible to approximate a variety of phenotypic traits and track their development over time using our previous work [4]. We show in Fig 13 an example of tracking traits such as leaf area and leaf length as well as traits related to the stem such as stem length and stem diameter. To evaluate our results, we compute the leaf lengths manually from the 3D point clouds similar to Golbach et al. [1] and observe a mean error of 22.4 mm for the maize plants and 8.9 mm for the tomato plants. Potentially, further methods to derived other traits can be derived from this dataset. However, given the aforementioned limitation in surface reconstruction from plant point clouds, the measurements of phenotypic traits are still an approximation based on a skeleton structure similar to Fig 11. In our previous work [4], after the segmentation and the skeletonization, we assign each point in the point cloud to the closest node in the skeleton. In this way, we obtain a fine-grained clusterization of each organ in small sub-regions. We then compute the main axis, or the main plane depending on the desired trait, of each sub-region using the standard singular value decomposition (SVD) approach. In this way, we can better estimate the shape of each sub-regions and derive phenotypic traits by summing the contribution of each sub-region. Note that different traits can be estimated from the skeleton structure of the plants. For example, we could estimate the axil for each leaf, i.e. the angle between the upper part of each leaf and the stem, using a similar procedure to the one described above, and track such trait over time. Thus, we believe that this dataset could also help to estimate a variety of phenotypic traits and to better understand their dynamics over time.

## 5 Summary

With this dataset paper, we release a unique agricultural dataset featuring 3D point clouds of plants recorded daily over a period of 2 weeks. Together with the raw point clouds, we provide the label for each point in the dataset for the task of organ segmentation. In total, we release 7 tomato plants recorded over three weeks, resulting in 140 distinct point clouds, and 7 maize plants recorded over two weeks, resulting in 84 point clouds (for the maize we provide 2 different annotation types, one based on the leaf collar method, the other based on the leaf tip method). To access and visualize the data we also provide a small set of APIs, featuring loading, visualization, and downsampling functionalities. This unprecedented effort in the agricultural domain results in a dataset containing a total of 126 labeled point clouds with approximately 260 million labeled 3D points that we are sharing with the community.

## 6 Future work

By making Pheno4D together with its software API publicly available and readily accessible online, we enable the efficient distribution of our data. We hope that Pheno4D will become an important resource for a broad range of computer vision and phenotyping research. Furthermore, we believe that the high accuracy and the extent of our dataset will enable it to become a new and challenging benchmark dataset for future research. While the current dataset already offers high standards in terms of the number of plants, the numbers of measuring dates and the point density and accuracy, there is still room for improvement. Background information, such as genotypes, phenology, management, nutrient availability or growing conditions may enhance the data and help to derive even more complex phenotypic traits. Also other crops might be of interest, as well stepping out of the green house into field conditions. Pheno4D does not claim to be a finished product but rather a dataset that will be improved and extended in the future. In addition, we gladly incorporate contributions from the research community, for example phenotyping results, in our dataset.
